# Existence of a scaling relation in continuous cultures of *Scheffersomyces stipitis*: the steady states are completely determined by the ratio of carbon and oxygen uptake rates

**DOI:** 10.1186/s13068-019-1357-3

**Published:** 2019-01-28

**Authors:** Shraddha Maitra, Atul Narang

**Affiliations:** Department of Biochemical Engineering and Biotechnology, Indian Institute of Technology Delhi, Hauz Khas, New Delhi, 110016 India

**Keywords:** Parametric sensitivity of ethanol production, Carbon limitation, Oxygen limitation, Dual limitation, *Scheffersomyces* (*Pichia*) *stipitis*

## Abstract

**Background:**

Recently, we showed that steady-state continuous cultures of *S.* *stipitis* follow the principles of growth on mixture of two complementary substrates. More precisely, when such cultures are fed with progressively higher concentrations of glucose *s*_f_ at fixed dilution rate *D* = 0.1 h^−1^, oxygen mass-transfer coefficient *k*_l_*a* = 50 h^−1^, and oxygen solubility $c_{\text{o}}^{*}$, they transition from glucose- to oxygen-limited growth through an intermediate dual-limited regime in which both glucose and oxygen are limiting, and ethanol is produced without loss of glucose. It is, therefore, of considerable interest to characterize the dual-limited regime. We found that the dual-limited regime occurs precisely when the operating parameters *D*, *s*_f_, *k*_l_*a,* and $c_{\text{o}}^{*}$ satisfy the relation $Y_{\text{os}} < Ds_{\text{f}} /\left( {k_{\text{l}} a \cdot c_{\text{o}}^{*} } \right) < Y_{\text{os}}^{\prime }$, where *Y*_os_ and $Y_{\text{os}}^{\prime }$ denote g of glucose consumed per g of oxygen consumed in the carbon- and oxygen-limited regimes. In this work, our goal was to determine if the above characterization of the dual-limited regime holds over a wider range of *D*, *k*_l_*a*, and to understand why the dual-limited regime is determined by the dimensionless ratio $Ds_{\text{f}} /\left( {k_{\text{l}} a \cdot c_{\text{o}}^{*} } \right)$.

**Results:**

To this end, we performed the foregoing experiments at three additional dilution rates (*D* = 0.07, 0.15, and 0.20 h^−1^) and one additional mass-transfer coefficient (*k*_l_*a* = 100 h^−1^). We find that the above characterization of the dual-limited regime is valid for these conditions as well. Furthermore, the boundaries of the dual-limited regime are determined by the dimensionless ratio $Ds_{\text{f}} /\left( {k_{\text{l}} a \cdot c_{\text{o}}^{*} } \right)$, because the steady-state concentrations are completely determined by this ratio. More precisely, if the steady-state concentrations of biomass, glucose, oxygen, and ethanol are suitably scaled, they collapse into a single curve with $Ds_{\text{f}} /\left( {k_{\text{l}} a \cdot c_{\text{o}}^{*} } \right)$ as the independent variable.

**Conclusion:**

The dual-limited regime is characterized by the relation $Y_{\text{os}} < Ds_{\text{f}} /\left( {k_{\text{l}} a \cdot c_{\text{o}}^{*} } \right) < Y_{\text{os}}^{{\prime }}$ over the entire range of operating condition 0.07 h^−1^ ≤ *D* ≤ 0.20 h^−1^ and $50 \;{\text{h}}^{ - 1} \le k_{\text{l}} a \le 100\;{\text{h}}^{ - 1}$. Since the effect of all operating parameters is embedded in the single parameter $Ds_{\text{f}} /\left( {k_{\text{l}} a \cdot c_{\text{o}}^{*} } \right)$, the dimensionless plot provides a powerful tool to compare, with only a handful of data, various ethanol-producing strains over a wide range of operating conditions.

**Electronic supplementary material:**

The online version of this article (10.1186/s13068-019-1357-3) contains supplementary material, which is available to authorized users.

## Background

Pretreatment of lignocellulosic biomass yields glucose and various pentoses [1]. Simultaneous consumption of hexoses and pentoses is therefore essential for one-step fermentation of sugars derived from lignocellulosic biomass. Among the best pentose-fermenting microbes are the yeasts *Candida shehatae*, *Pachysolen tannophilus,* and *Scheffersomyces stipitis* (referred to earlier as *Pichia stipitis* [2]). *S. stipitis* is of particular interest, because it gives a high ethanol yield, produces almost no by-products, and requires minimal addition of vitamins to stimulate fermentation [3–5].

In spite of the foregoing desirable properties, *S. stipitis* is not the organism of choice for large-scale ethanol production. This is primarily because it grows and ferments only in a narrow range of dissolved oxygen concentrations: under aerobic conditions, it grows but does not ferment, and under anaerobic conditions, it ferments but grows poorly [6–9]. This has led to the conclusion that production of ethanol by *S. stipitis* is not robust and, therefore, unsuitable for large-scale production [10, 11].

Although ethanol production by *S. stipitis* is not robust, it would be useful to quantify this in a manner that is useful for systematic process and strain improvement. This could be done by growing the cells in a chemostat and determining the parametric sensitivity of ethanol production, i.e., the range of the operating parameters (rather than dissolved oxygen concentrations) that support ethanol production. The key operating parameters for the system are the dilution rate *D*, the feed concentration of the carbon source *s*_f_, the mass-transfer coefficient of oxygen *k*_l_*a* (determined primarily by the stirrer speed and gas flow rate), and the solubility of oxygen in the aqueous medium $c_{\text{o}}^{*}$ (determined primarily by the oxygen content of the gaseous stream). Although there are several studies of ethanol production by continuous cultures of *S. stipitis* [8, 12–15], the parametric sensitivity of ethanol production has not been quantified.

Recently, we reported a method for quantifying the parametric sensitivity of ethanol production by *S. stipitis* [15]. Our method was based on the principles that govern the manner in which a culture transitions from growth limited on one substrate to another substrate that is complementary to it. Examples include the transition from carbon- to nitrogen- or phosphorus- or magnesium-limited growth. These transitions are generally studied by feeding steady-state continuous cultures with progressively higher concentrations of the carbon source while keeping the feed concentrations of all other nutrients at a constant value [16, 17], although the same information can also be obtained by changing the feed concentration slowly [18]. Under these conditions, the culture undergoes a transition from carbon-limited growth at low feed concentrations of the carbon source to limitation by another nutrient at high feed concentrations of the carbon source [19]. It was widely believed, since the time of Liebig that this transition occurred abruptly, i.e., there existed a feed concentration of the carbon source at which the culture switched abruptly from carbon limitation to limitation by another nutrient. In 1991, Egli analyzed the data from a wide variety of papers, and showed that the transition did not occur abruptly [19]. There was a range of feed concentrations over which the culture was limited by both nutrients, and both nutrients were completely consumed in this dual-limited regime.

In our recent work, we showed that the transition from carbon to oxygen limitation also follows the above-mentioned pattern of growth even though oxygen is an electron acceptor rather than a nutrient [15]. Specifically, we showed that if the concentration of glucose fed to a continuous culture of *S. stipitis* was progressively increased at fixed *D* = 0.1 h^−1^ and *k*_l_*a* ≈ 50 h^−1^, the resultant steady states showed three distinct and well-defined growth regimes. At low feed concentrations of glucose ($s_{\text{f}} < \underline {s}_{\text{f}}$), growth was carbon-limited, dissolved oxygen was in excess, and no ethanol was produced. At high feed concentrations of glucose ($s_{\text{f}} > \bar{s}_{\text{f}}$), growth was oxygen-limited and ethanol was produced, but the residual glucose concentration was high and led to significant loss of unused glucose. At intermediate feed concentrations of glucose ($\underline{s}_{\text{f}} < s_{\text{f}} < \bar{s}_{\text{f}}$), growth was dual-limited, i.e., both glucose and oxygen were limiting, and ethanol was produced without loss of glucose. Thus, we found that at fixed *D* and *k*_l_*a*, it is desirable to operate the chemostat in the dual-limited regime $\underline{s}_{\text{f}} < s_{\text{f}} < \bar{s}_{\text{f}}$. We then developed simple unstructured mathematical models to understand what determines the boundaries, $\underline{s}_{\text{f}}$ and $\bar{s}_{\text{f}}$, of the dual-limited regime. The models yielded the expressions $\underline{s}_{\text{f}} = Y_{\text{os}} k_{\text{l}} a \cdot c_{\text{o}}^{*} /D$ and $\bar{s}_{\text{f}} = Y_{\text{os}}^{{\prime }} k_{\text{l}} a \cdot c_{\text{o}}^{*} /D$, where *Y*_os_ and $Y_{\text{os}}^{{\prime }}$ denote g of glucose consumed per g of oxygen consumed in the carbon- and oxygen-limited regimes, respectively. The boundaries predicted by these expressions agreed well with the boundaries observed in our experiments performed at *D* = 0.1 h^−1^ and *k*_l_*a* ≈ 50 h^−1^. Analysis of the model also showed that the chemostat is in the dual-limited regime (and ethanol is produced without loss of glucose) precisely when the key operating parameters are such that $Y_{\text{os}} < Ds_{\text{f}} /\left( {k_{\text{l}} a \cdot c_{\text{o}}^{*} } \right) < Y_{\text{os}}^{\prime }$, i.e., the dual-limited regime is completely determined by the dimensionless ratio $Ds_{\text{f}} /\left( {k_{\text{l}} a \cdot c_{\text{o}}^{*} } \right)$.

In this work, our first goal was to test the validity of the above expressions for $\underline{s}_{\text{f}}$ and $\bar{s}_{\text{f}}$ over a range of *D* and *k*_l_*a*. To this end, we performed the foregoing experiment at three additional dilution rates (0.07, 0.15, and 0.20 h^−1^) and an additional mass-transfer coefficient (100 h^−1^). We found that the values of $\underline{s}_{\text{f}}$ and $\bar{s}_{\text{f}}$ decreased with *D* and increased with *k*_l_*a* in a manner consistent with the above expressions. Our second goal was to understand why the dual-limited regime is completely determined by the dimensionless ratio $Ds_{\text{f}} /\left( {k_{\text{l}} a \cdot c_{\text{o}}^{*} } \right)$. We found that this occurs because the steady-state concentrations of biomass, residual glucose, dissolved oxygen, and ethanol, when suitably scaled, are completely determined by $Ds_{\text{f}} /\left( {k_{\text{l}} a \cdot c_{\text{o}}^{*} } \right)$. In other words, when the data obtained at various *D* and *k*_l_*a* are suitably scaled and plotted against $Ds_{\text{f}} /\left( {k_{\text{l}} a \cdot c_{\text{o}}^{*} } \right)$, every measured concentration collapses into a single curve. We show that this dimensionless plot provides a powerful tool for comparing, without significant data acquisition, the performance of different strains over a wide range of operating conditions.

### Model for carbon- and oxygen-limited growth of *S. stipitis* in a chemostat

In our continuous culture experiments, we measured the steady-state concentrations of biomass (*x*), residual glucose (*s*), dissolved oxygen (*c*_o_), and ethanol (*p*) obtained when *s*_f_ was varied at fixed *D*, *k*_l_*a*, and $c_{\text{o}}^{*}$. These steady-state concentrations satisfy the mass balance equations:

1 $$0 = \frac{{{\text{d}}x}}{{{\text{d}}t}} = - Dx + \mu x,$$

2 $$0 = \frac{{{\text{d}}s}}{{{\text{d}}t}} = D\left( {s_{\text{f}} - s} \right) - r_{\text{s}} x,$$

3 $$0 = \frac{{{\text{d}}c_{\text{o}} }}{{{\text{d}}t}} = k_{\text{l}} a\left( {c_{\text{o}}^{*} - c_{\text{o}} } \right) - r_{\text{o}} x,$$

4 $$0 = \frac{{{\text{d}}p}}{{{\text{d}}t}} = - Dp + r_{\text{p}} x,$$

where *μ*, *r*_s_, *r*_o_, and *r*_p_ represent the specific rates of biomass growth, glucose consumption, oxygen consumption, and ethanol formation, respectively. It follows that the non-trivial steady states (*x* > 0) satisfy the equations:

5 $$\mu = D,$$

6 $$D(s_{\text{f}} - s) = r_{\text{s}} x,$$

7 $$k_{\text{l}} a\left( {c_{\text{o}}^{*} - c_{\text{o}} } \right) = r_{\text{o}} x,$$

8 $$Dp = r_{\text{p}} x,$$

which will provide the steady-state concentrations after we specify the rates *μ*, *r*_s_, *r*_o_, and *r*_p_.

#### Steady states of carbon-limited regime

We assume that carbon-limited growth can be described by the single quasi-reaction:

9 $$Y_{\text{xs}} {\text{S + }} Y_{\text{xo}} {{\text{O}}_{2}} \mathop \to \limits^{\mu \left( s \right)} {\text{X }} + Y_{\text{xp}} {\text{P }} + Y_{\text{xc}} {{\text{CO}}_{2}} ,\qquad \mu \left( s \right) = \mu_{\text{m}} \frac{s}{{K_{\text{s}} + s}},$$

where *μ*(*s*) is the specific growth rate, *μ*_m_ is the maximum specific growth rate, and *K*_s_ is the saturation constant. It follows that the specific rates of glucose consumption, oxygen consumption, and ethanol formation are given by the expressions:

10 $$r_{\text{s}} = Y_{\text{xs}} \mu \left( s \right) = \frac{\mu \left( s \right)}{{Y_{\text{sx}} }},$$

11 $$r_{\text{o}} = Y_{\text{xo}} \mu \left( s \right) = \frac{\mu \left( s \right)}{{Y_{\text{ox}} }},$$

12 $$r_{\text{p}} = Y_{\text{xp}} \mu \left( s \right) = \frac{\mu \left( s \right)}{{Y_{\text{px}} }},$$

where *Y*_sx_, *Y*_ox_, and *Y*_px_ are the yields of biomass on glucose, oxygen, and ethanol, respectively.

If the dilution rate is sufficiently small, almost all the substrate entering the reactor is consumed. Substituting (9)–(12) in (5)–(8) then yields

13 $$s = K_{\text{s}} \frac{D}{{\mu_{\text{m}} - D}} \ll s_{\text{f}} ,$$

14 $$x = Y_{\text{sx}} \left( {s_{\text{f}} - s} \right) \approx Y_{\text{sx}} s_{\text{f}} ,$$

15 $$p = Y_{\text{sp}} \left( {s_{\text{f}} - s} \right) \approx Y_{\text{sp}} s_{\text{f}} ,$$

16 $$\frac{{c_{\text{o}} }}{{c_{\text{o}}^{*} }} = 1 - \frac{{\left( {D/Y_{\text{ox}} } \right)x}}{{k_{\text{l}} a \cdot c_{\text{o}}^{*} }} \approx 1 - \left( {\frac{D}{{Y_{\text{os}} k_{\text{l}} a \cdot c_{\text{o}}^{*} }}} \right)s_{\text{f}} ,$$

where *Y*_sp_ ≡ *Y*_sx_/*Y*_px_ denotes g of ethanol produced per g of glucose and *Y*_os_ ≡ *Y*_ox_/*Y*_sx_ denotes g of glucose consumed per g of oxygen. It follows from Eq. (16) that if *s*_f_ is increased at a fixed *D*, *k*_l_*a*, and $c_{\text{o}}^{*}$, the dissolved oxygen concentration decreases linearly becoming zero at

17 $$s_{\text{f}} = \underline{s}_{\text{f}} \equiv \frac{{Y_{\text{os}} k_{\text{l}} a \cdot c_{\text{o}}^{*} }}{D},$$

which provides a good approximation to the feed concentration at which the cells transition from carbon- to dual-limited growth. It should be noted that in reality, *c*_o_ does not become zero at the transition point $s_{\text{f}} = \underline{s}_{\text{f}}$. Instead, it reaches sub-critical levels, which are so small that *c*_o_ = 0 is a good approximation to the concentration at the transition point.

#### Steady states of oxygen-limited regime

We assume that oxygen-limited growth can be described by the single quasi-reaction:

18 $$Y_{\text{xs}}^{\prime } {\text{S + }}Y_{\text{xo}}^{\prime } {\text{O}}_{2} \mathop \to \limits^{{\mu^{\prime}\left( {c_{\text{o}} } \right)}} {\text{X }} + Y_{\text{xp}}^{\prime } {\text{P }} + Y_{\text{xc}}^{\prime } {\text{CO}}_{2} , \;\;\;\mu^{\prime}\left( {c_{\text{o}} } \right) = \mu_{\text{m}} \frac{{c_{\text{o}} }}{{K_{\text{o}} + c_{\text{o}} }},$$

where $\mu$^′^(*c*_o_) denotes the specific growth rate, and *K*_o_ denotes the critical dissolved oxygen level. It follows that the specific rates of glucose consumption, oxygen consumption, and ethanol production are given by the expressions:

19 $$r_{\text{s}}^{\prime } = Y_{\text{xs}}^{\prime } \mu^{\prime}\left( {c_{\text{o}} } \right) = \frac{{\mu^{\prime}\left( {c_{\text{o}} } \right)}}{{Y_{\text{sx}}^{\prime } }},$$

20 $$r_{\text{o}}^{\prime } = Y_{\text{xo}}^{\prime } \mu^{\prime}\left( {c_{\text{o}} } \right) = \frac{{\mu^{\prime}\left( {c_{\text{o}} } \right)}}{{Y_{\text{ox}}^{\prime } }},$$

21 $$r_{\text{p}}^{\prime } = Y_{\text{xp}}^{\prime } \mu^{\prime}\left( {c_{\text{o}} } \right) = \frac{{\mu^{\prime}\left( {c_{\text{o}} } \right)}}{{Y_{\text{px}}^{\prime } }},$$

where $Y_{\text{sx}}^{\prime }$, $Y_{\text{ox}}^{\prime }$, and $Y_{\text{px}}^{\prime }$ are the yields of biomass on substrate, oxygen, and product under oxygen-limited conditions.

If the dilution rate is sufficiently small, the dissolved oxygen level is small compared to saturating levels. Substituting (18)–(21) in (5)–(8) then yields

22 $$\frac{{c_{\text{o}} }}{{c_{\text{o}}^{*} }} = \frac{{K_{\text{o}} }}{{c_{\text{o}}^{*} }}\frac{D}{{\left( {\mu_{\text{m}} - D} \right)}} \ll 1,$$

23 $$x = \frac{{Y_{\text{ox}}^{\prime } k_{\text{l}} a\left( {c_{\text{o}}^{*} - c_{\text{o}} } \right)}}{D} \approx \frac{{Y_{\text{ox}}^{\prime } k_{\text{l}} a \cdot c_{\text{o}}^{*} }}{D},$$

24 $$p = \frac{{Y_{\text{op}}^{\prime } k_{\text{l}} a\left( {c_{\text{o}}^{*} - c_{\text{o}} } \right)}}{D} \approx \frac{{Y_{\text{op}}^{\prime } k_{\text{l}} a \cdot c_{\text{o}}^{*} }}{D},$$

25 $$s = s_{\text{f}} - \frac{{Y_{\text{os}}^{\prime } k_{\text{l}} a\left( {c_{\text{o}}^{*} - c_{\text{o}} } \right)}}{D} \approx s_{\text{f}} - \frac{{Y_{\text{os}}^{\prime } k_{\text{l}} a \cdot c_{\text{o}}^{*} }}{D},$$

where $Y_{\text{op}}^{\prime } \equiv {{Y_{\text{ox}}^{\prime } } \mathord{\left/ {\vphantom {{Y_{\text{ox}}^{\prime } } {Y_{\text{px}}^{\prime } }}} \right. \kern-0pt} {Y_{\text{px}}^{\prime } }}$ denotes the g of ethanol produced per g of oxygen consumed, and $Y_{\text{os}}^{\prime } \equiv {{Y_{\text{ox}}^{\prime } } \mathord{\left/ {\vphantom {{Y_{\text{ox}}^{\prime } } {Y_{\text{sx}}^{\prime } }}} \right. \kern-0pt} {Y_{\text{sx}}^{\prime } }}$ denotes the g of glucose consumed per g of oxygen consumed. It follows from Eq. (25) that if *s*_f_ is increased at fixed *D*, *k*_l_*a*, and $c_{\text{o}}^{*}$, the concentration of residual glucose increases linearly. Letting *s* = 0 in Eq. (25) yields

26 $$s_{\text{f}} = \bar{s}_{\text{f}} \equiv \frac{{Y_{\text{os}}^{\prime } k_{\text{l}} a \cdot c_{\text{o}}^{*} }}{D},$$

which provides a good approximation to the feed concentration of glucose at which the cells transition from the dual-limited to the oxygen-limited regime.

## Results

### The concentration profiles are qualitatively similar at all *D* and *k*_l_*a*

**Fig. 1:**
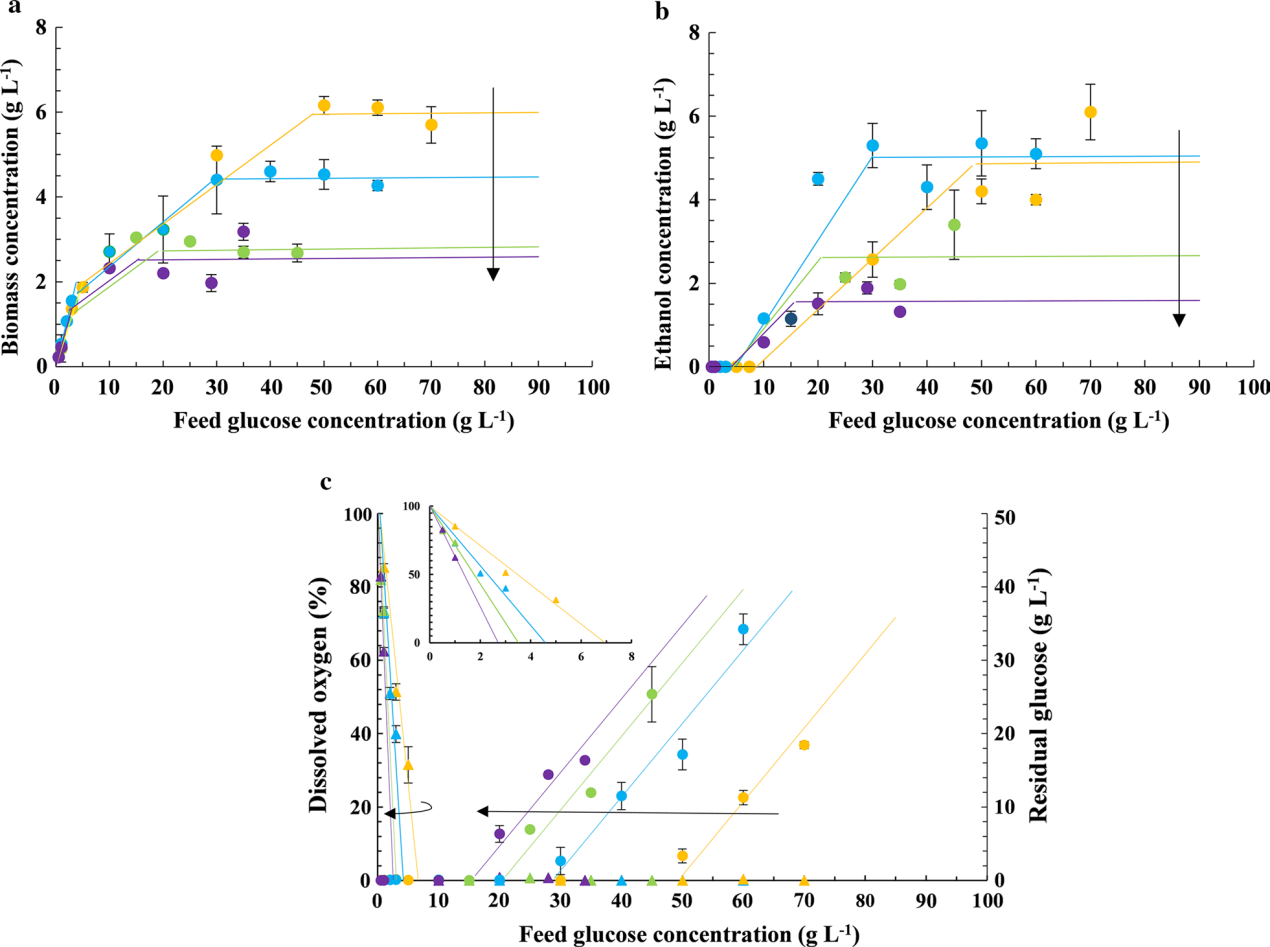
Variation of the steady-state concentrations of **a** biomass, **b** ethanol, **c** dissolved oxygen and residual glucose with respect to the feed concentration of glucose at fixed *k*_l_*a* ≈ 50 h^−1^ and four dilution rates, namely, 0.07 h^−1^ (yellow), 0.10 h^−1^ (blue), 0.15 h^−1^ (green), and 0.20 h^−1^ (violet). The arrows point in the direction of increasing *D*. The lines in the carbon- and oxygen-limited regimes are best fits to Eqs. (14)–(16) and (23)–(25), respectively. The lines in the dual-limited regime are obtained by linearly interpolating the end points of the carbon- and oxygen-limited regimes. The inset in **c** shows the dissolved oxygen levels

**Fig. 2:**
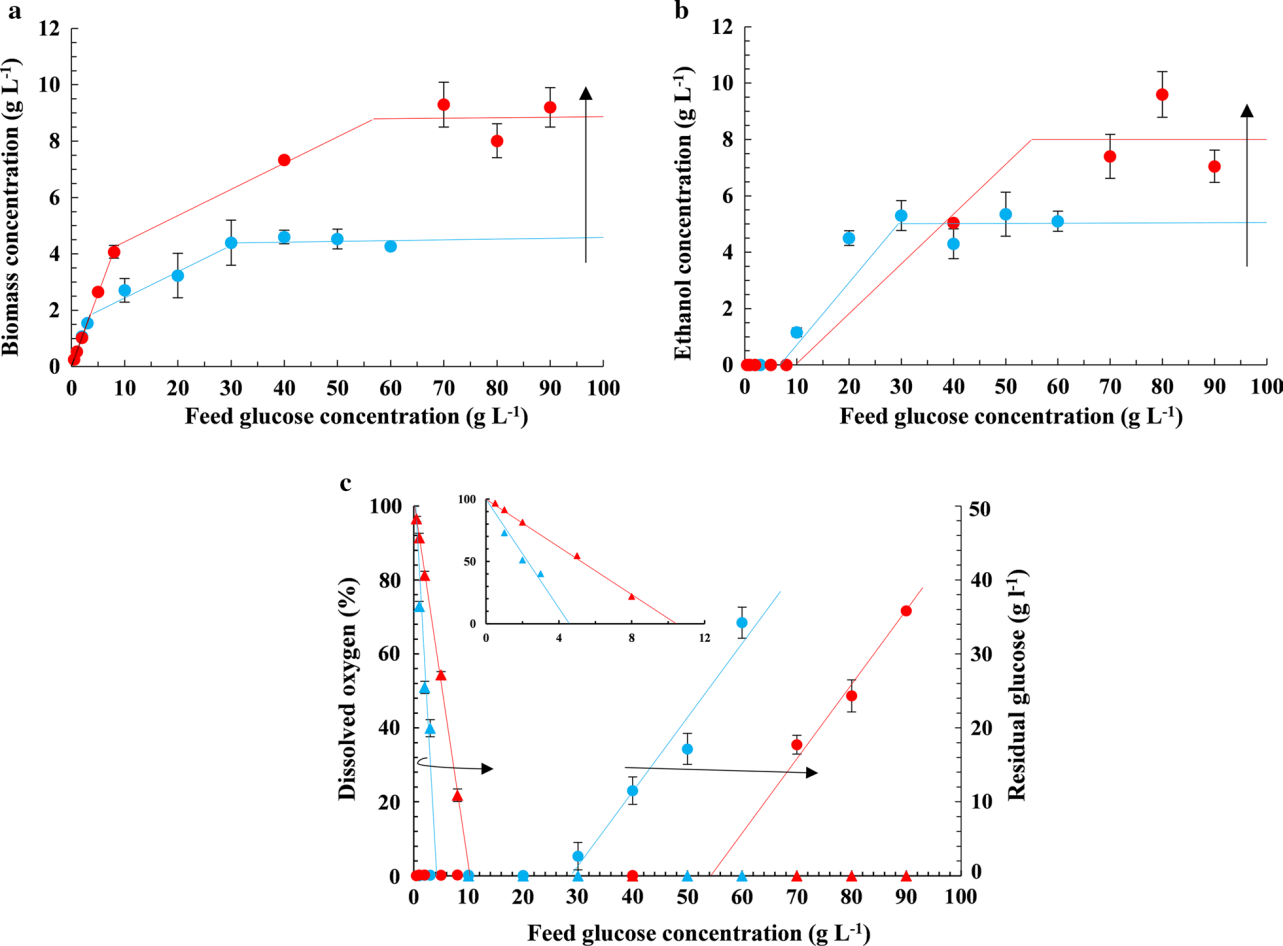
Variation of the steady-state concentrations of **a** biomass, **b** ethanol, **c** dissolved oxygen and residual glucose with the feed concentration of glucose at fixed *D* = 0.1 h^−1^ and two mass-transfer coefficients, namely, *k*_l_*a* ≈ 50 h^−1^ (blue) and *k*_l_*a* ≈ 100 h^−1^ (red). The arrows point in the direction of increasing *k*_l_*a*. The lines in the carbon- and oxygen-limited regimes represent best fits to Eqs. (14)–(16) and (23)–(25), respectively. The lines in the dual-limited regime are obtained by linearly interpolating the end points of the carbon- and oxygen-limited regimes. The inset in **c** shows the dissolved oxygen levels

Recently, we showed that if the concentration of glucose fed to a chemostat was increased at fixed *D* = 0.1 h^−1^ and *k*_l_*a* ≈ 50 h^−1^, cultures of *S. stipitis* transitioned from carbon- to oxygen-limited growth via an intermediate dual-limited regime [20]. In this work, our goal was to study the effect of *D* and *k*_l_*a* on the steady-state concentration profiles. To this end, we repeated the foregoing continuous culture studies by (a) varying *D* ($D = 0.07, \;\;0.15,\;\; 0.20$ h^−1^) at fixed *k*_l_*a* ≈ 50 h^−1^ (Fig. 1), and (b) varying *k*_l_*a* ($k_{\text{l}} a \approx 50, 100$ h^−1^) at fixed $D = 0.1$ h^−1^ (Fig. 2). As we show below, there were three distinct and well-defined growth regimes in all the experiments, and the concentration profiles in each regime were qualitatively similar.

At low feed concentrations of glucose, growth was carbon-limited. Under these conditions, no glucose and ethanol were detected in the effluent stream. When the feed concentration of glucose was increased, the biomass concentration increased linearly, and the dissolved oxygen level decreased linearly until it became near-zero at a sufficiently large value of the glucose feed concentration denoted $\underline{s}_{\text{f}}$.

When the feed concentration of glucose was increased beyond $\underline{s}_{\text{f}}$, growth was both carbon- and oxygen-limited. In this dual-limited regime, the dissolved oxygen levels were immeasurably low, which implies that the oxygen transfer rate $k_{\text{l}} a \cdot \left( {c_{\text{o}}^{*} - c_{\text{o}} } \right)$ had reached the maximum (mass-transfer-limited) level $k_{\text{l}} a \cdot c_{\text{o}}^{*}$. Yet, additional glucose fed to the chemostat was completely consumed and led to the synthesis of additional biomass. The additional glucose was presumably channeled into fermentation, since its consumption was accompanied by production of ethanol. The dual-limited regime persisted until the glucose feed concentration increased to sufficiently large value denoted $\bar{s}_{\text{f}}$.

When the glucose concentration in the feed was increased beyond $\bar{s}_{\text{f}}$, dissolved oxygen levels remained undetectable and the residual glucose levels increased dramatically, which indicates that growth was now oxygen-limited. In this regime, ethanol and biomass concentrations remained constant, but the effluent glucose levels increased linearly with the glucose feed concentration. This suggests that glucose uptake had saturated in this regime—the additional glucose supplied simply exited the reactor without any consumption by the cells.

Although the concentration profiles were qualitatively similar at all *D* and *k*_l_*a*, there were quantitative differences. In what follows, we shall describe these differences and show that they can be explained in terms of the model. However, before doing so, it is necessary to check the validity of the model.

### The yields observed in carbon- and oxygen-limited growth are nearly constant

Our models of carbon- and oxygen-limited growth are based on two assumptions:

1. The specific growth rates during carbon- and oxygen-limited growth, denoted *μ* and *μ*^′^, are functions of the glucose and dissolved oxygen concentrations, respectively.
2. The biomass yields during carbon- and oxygen-limited growth, denoted *Y*_sx_, *Y*_ox_, *Y*_px_ and $Y_{\text{sx}}^{\prime }$, $Y_{\text{ox}}^{\prime }$, $Y_{\text{px}}^{\prime }$, respectively, are constant.

The validity of the first assumption was demonstrated above: in the carbon-limited (resp. oxygen-limited) regime, the concentration of glucose (resp. dissolved oxygen) was immeasurably small, while that of dissolved oxygen (resp. glucose) was large. It remains to verify the validity of the second assumption.

Given the steady-state concentrations at various operating conditions (Figs. 1, 2), the steady-state mass balance equations (1)–(4) can be used to calculate the corresponding specific rates of glucose uptake, oxygen consumption, and ethanol formation. In the carbon- and oxygen-limited regimes, these rates are independent of the glucose feed concentration and the oxygen mass-transfer coefficient (Additional file 1: Figure S1), and increase linearly with the dilution rate (Additional file 1: Figure S2), which in turn equals the specific growth rate. It follows that the yields in the carbon-limited regime (*Y*_sx_, *Y*_ox_, *Y*_px_) and oxygen-limited regime ($Y_{\text{sx}}^{\prime }$, $Y_{\text{ox}}^{\prime }$, $Y_{\text{px}}^{\prime }$) are essentially constant (Table 1).

**Table 1:** Yields obtained during carbon- and oxygen-limited growth of continuous cultures of *S. stipitis*. The biomass yields shown in rows 1–3 were obtained by fitting the data (Additional file 1: Figure S2), and were used to calculate the remaining yields shown in rows 4–5, which appear in the dimensionless Eqs. (36), (37)

Carbon-limited regime		Oxygen-limited regime	
*Y*_sx_	0.44 g g^−1^	$Y_{\text{sx}}^{\prime }$	0.15 g g^−1^
*Y*_px_	∞	$Y_{\text{px}}^{\prime }$	1.11 g g^−1^
*Y*_ox_	0.55 g g^−1^	$Y_{\text{ox}}^{\prime }$	0.91 g g^−1^
*Y*_os_ = *Y*_ox_/*Y*_sx_	1.25 g g^−1^	$Y_{\text{os}}^{\prime } = {{Y_{\text{ox}}^{\prime } } \mathord{\left/ {\vphantom {{Y_{\text{ox}}^{\prime } } {Y_{\text{sx}}^{\prime } }}} \right. \kern-0pt} {Y_{\text{sx}}^{\prime } }}$	6.06 g g^−1^
*Y*_op_ = *Y*_ox_/*Y*_px_	0	$Y_{\text{op}}^{\prime } = {{Y_{\text{ox}}^{\prime } } \mathord{\left/ {\vphantom {{Y_{\text{ox}}^{\prime } } {Y_{\text{px}}^{\prime } }}} \right. \kern-0pt} {Y_{\text{px}}^{\prime } }}$	0.82 g g^−1^

Since both assumptions of our model are valid, we can proceed to analyze the data in terms of the model. Before doing so, however, we note that

27 $$Y_{\text{sx}} > Y_{\text{sx}}^{\prime } , \;\;Y_{\text{ox}}^{\prime } > Y_{\text{ox}} .$$

That is, the amount of biomass produced per g of glucose is higher when carbon is limiting, and the amount of biomass produced per g of oxygen is higher when oxygen is limiting. In other words, the efficiency of biomass synthesis with respect to a particular factor is highest when that factor is limiting. Equation (27) then implies that $Y_{\text{os}} < Y_{\text{os}}^{\prime }$, and it follows from Eqs. (17) and (26), implying that $\underline{s}_{\text{f}} < \bar{s}_{\text{f}}$.

### The variation of the concentration profiles with $D$ and $k_{\text{l}} a$ is consistent with the model

Our next goal is to explain, in terms of the model, the variation of the concentration profiles with *s*_f_ for various *D* and *k*_l_*a* ($c_{\text{o}}^{*}$ was not varied, since we used the air in all the experiments).

#### Carbon-limited regime

In this regime, the cell density increases linearly with the feed concentration of glucose regardless of *D* and *k*_l_*a* (Figs. 1a, 2a), whereas the ethanol concentration is always negligible. This is consistent with Eqs. (14), (15), which reflect the fact that at steady state, the biomass and ethanol efflux rates *Dx*, *Dp* equal the respective biomass and ethanol generation rates *μx*, *r*_p_*x* which, under carbon-limited conditions, are proportional to the maximum glucose consumption rate *Ds*_f_, that is

28 $$Dx = \mu x = \left( {Y_{\text{sx}} r_{\text{s}} } \right)x = Y_{\text{sx}} \cdot D\left( {s_{\text{f}} - s} \right) \approx Y_{\text{sx}} \cdot Ds_{\text{f}} .$$

29 $$Dp = r_{\text{p}} x = \left( {Y_{\text{sp}} r_{\text{s}} } \right)x = Y_{\text{sp}} \cdot D\left( {s_{\text{f}} - s} \right) \approx Y_{\text{sp}} \cdot Ds_{\text{f}} .$$

It follows from (28) that *x* is proportional to *s*_f _—it is independent of *k*_l_*a* since the biomass efflux and generation rates are independent of *k*_l_*a*, and independent of *D* since both rates are proportional to *D*. Equation (29) implies that *p* = 0 since Crabtree-negative yeasts do not ferment under aerobic conditions (*Y*_sp_ = 0).

At fixed *D* and *k*_l_*a*, the dissolved oxygen concentration decreases linearly with the feed concentration (Figs. 1c, 2c). The linear dissolved oxygen concentration profile rotates about the point (0, 100) clockwise if *D* is increased at fixed *k*_l_*a* (Fig. 1c) and counter-clockwise if *k*_l_*a* is increased at fixed *D* (Fig. 2c). These trends are consistent with Eq. (16) which follows from the fact that at steady state, the oxygen transfer rate *k*_l_*a* ($c_{\text{o}}^{*}$ − *c*_o_) equals the oxygen consumption rate *r*_o_*x*, which, under carbon-limited conditions, is proportional to the maximum glucose consumption rate *Ds*_f_, that is

30 $$k_{\text{l}} a\left( {c_{\text{o}}^{*} - c_{\text{o}} } \right) = r_{\text{o}} x = \left( {Y_{\text{so}} r_{\text{s}} } \right)x = Y_{\text{so}} \cdot D(s_{\text{f}} - s) \approx Y_{\text{so}} \cdot Ds_{\text{f}} .$$

It follows that if *s*_f_ is increased at fixed *D* and *k*_l_*a* (Figs. 1c, 2c), or *D* is increased at fixed *k*_l_*a* and *s*_f_ (Fig. 1c), the oxygen demand $Y_{\text{so}} \cdot Ds_{\text{f}}$ increases—due to enhanced biomass concentration *x* ≈ *Y*_sx_*s*_f_ in the first case and enhanced specific oxygen consumption rate (*r*_o_ = *DY*_xo_) in the second case—and since *k*_l_*a* is constant, the dissolved oxygen level decreases to meet the enhanced oxygen demand. Conversely, if *k*_l_*a* is increased at fixed *D* and *s*_f_ (Fig. 2c), the dissolved oxygen level increases, because *k*_l_*a* increases at constant oxygen demand.

#### Oxygen-limited regime

In the oxygen-limited regime, the biomass and ethanol concentrations are constant whenever *D* and *k*_l_*a* are fixed. However, these constant levels decline if *D* is increased at fixed *k*_l_*a* (Fig. 1a, b), and increase if *k*_l_*a* is increased at fixed *D* (Fig. 2a, b). These trends are consistent with Eqs. (23), (24) which follow from the fact that at steady state, the biomass and ethanol efflux rates, *Dx* and *Dp*, are equal to their respective production rates, *μx* and *r*_p_*x*, but under oxygen-limited conditions, both production rates are proportional to the maximum oxygen uptake rate $k_{\text{l}} a \cdot c_{\text{o}}^{*}$, that is

31 $$Dx = \mu x = \left( {Y_{\text{ox}}^{\prime } r_{\text{o}} } \right)x = Y_{\text{ox}}^{\prime } \cdot k_{\text{l}} a(c_{\text{o}}^{*} - c_{\text{o}} ) \approx Y_{\text{ox}}^{\prime } \cdot \left( {k_{\text{l}} a \cdot c_{\text{o}}^{*} } \right),$$

32 $$Dp = r_{\text{p}} x = \left( {Y_{\text{op}}^{\prime } r_{\text{o}} } \right)x = Y_{\text{op}}^{\prime } \cdot k_{\text{l}} a(c_{\text{o}}^{*} - c_{\text{o}} ) \approx Y_{\text{op}}^{\prime } \cdot \left( {k_{\text{l}} a \cdot c_{\text{o}}^{*} } \right).$$

It follows that *x* and *p* are constant at fixed *D* and *k*_l_*a*. However, when *D* is increased at fixed *k*_l_*a* (Fig. 1a, b), the biomass and ethanol concentrations decrease, because the effluent is removed at a faster rate, while the biomass and ethanol production rates, $Y_{\text{ox}}^{\prime } \left( {k_{\text{l}} a \cdot c_{\text{o}}^{*} } \right)$ and $Y_{\text{op}}^{\prime } \left( {k_{\text{l}} a \cdot c_{\text{o}}^{*} } \right)$, are fixed. Conversely, if *k*_l_*a* is increased at fixed *D* (Fig. 2a, b), the biomass and ethanol concentrations increase, because the biomass and ethanol production rates increase, while the effluent flow rate remains fixed.

At fixed *D* and *k*_l_*a*, the residual glucose concentration increases linearly with the feed concentration of glucose (Figs. 1c, 2c). However, the residual glucose concentration profile shifts up if *D* is increased at fixed *k*_l_*a* (Fig. 1c), and shifts down if *k*_l_*a* is increased at fixed *D* (Fig. 2c). These variations are consistent with Eq. (25) which ultimately expresses the fact that at steady state, the net glucose influx rate equals the glucose consumption rate, which under oxygen-limited conditions, is proportional to the maximum oxygen consumption rate $k_{\text{l}} a \cdot c_{\text{o}}^{*}$, that is

33 $$D\left( {s_{\text{f}} - s} \right) = r_{\text{s}} x = \left( {Y_{\text{os}}^{\prime } r_{\text{o}} } \right)x = Y_{\text{os}}^{\prime } \cdot k_{\text{l}} a(c_{\text{o}}^{*} - c_{\text{o}} ) \approx Y_{\text{os}}^{\prime } \cdot \left( {k_{\text{l}} a \cdot c_{\text{o}}^{*} } \right).$$

It follows when *D* and *k*_l_*a* are fixed, *s*_f_ − *s* is a constant equal to $\bar{s}_{\text{f}}$, i.e., *s* lies at a distance $\bar{s}_{\text{f}}$ vertically below the line *s* = *s*_f_. If *D* is increased at fixed *k*_l_*a* (Fig. 1c), *s*_f_ − *s* decreases, since the flow rate is increased, while the substrate consumption rate is constant; conversely, if *D* is increased at fixed *k*_l_*a* (Fig. 2c), *s*_f_ − *s* increases, since the substrate consumption rate increases, while the flow rate is constant.

### The variation of the growth boundaries with $D$ and $k_{\text{l}} a$ is consistent with the model

Our next goal is to compare the experimentally determined boundaries of dual-limited growth with the model predictions.

**Fig. 3:**
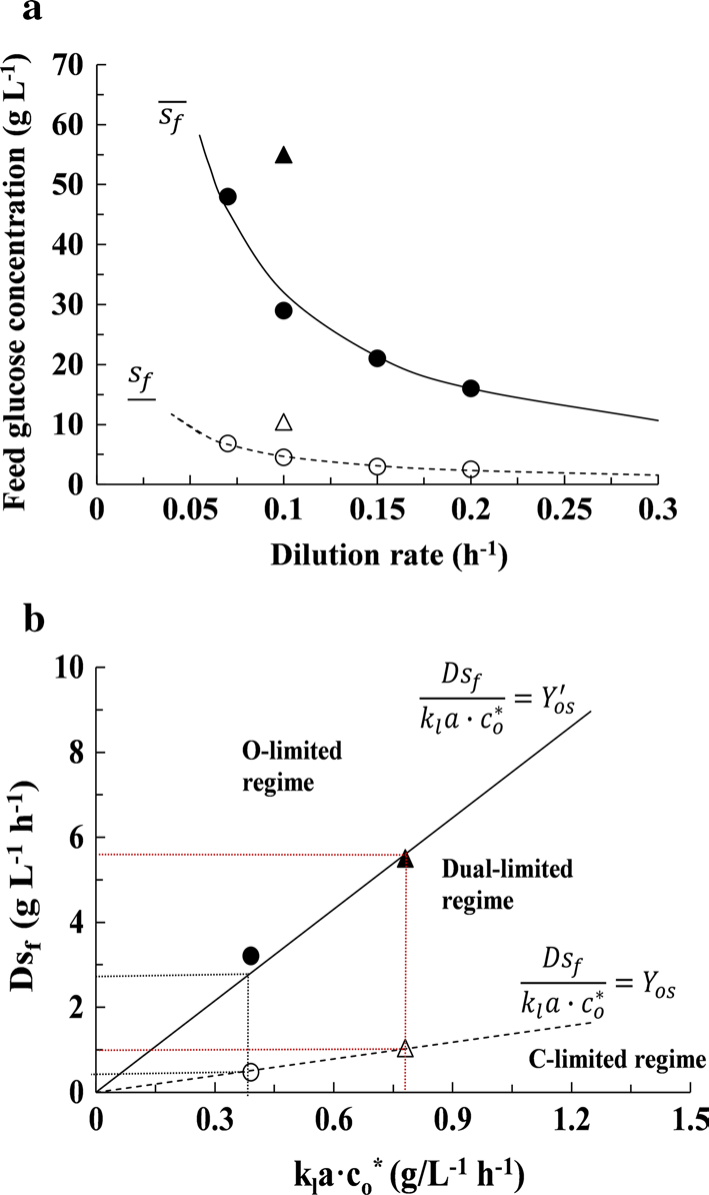
Variation of the boundaries of the dual-limited regime with the operating parameters. **a** At fixed *k*_l_*a* ≈ 50 h^−1^, the experimentally determined values of $\underline{s}_{\text{f}}$ (open circle) and $\bar{s}_{\text{f}}$ (closed circle) are inversely proportional to *D*. The dashed and solid curves, which represent the best fits to the data, satisfy the equations $D\underline{s}_{\text{f}}$ = 0.47 g L^−1^ h^−1^ and $D\bar{s}_{\text{f}}$= 3.2 g L^−1^ h^−1^, respectively. At fixed *D* = 0.10 h^−1^, the values of $\underline{s}_{\text{f}}$ and $\bar{s}_{\text{f}}$ at *k*_l_*a* ≈ 50 h^−1^, represented by open circle and closed circle, respectively, are half the values of $\underline{s}_{\text{f}}$ and $\bar{s}_{\text{f}}$ at *k*_l_*a* ≈ 100 h^−1^, represented by open triangle and closed triangle, respectively. **b** Representation of the data in **a** on the $k_{\text{l}} a \cdot c_{\text{o}}^{*} , Ds_{\text{f}}$-plane

For each set of the data sets in Figs. 1 and 2, we determined the lower and upper boundaries of dual-limited growth as follows. The lower boundary of the dual-limited regime $s_{\text{f}} = \underline{s}_{\text{f}}$ was determined by fitting the dissolved oxygen concentration profile in the carbon-limited regime to the one-parameter linear equation (16), and the upper boundary of the dual-limited regime $s_{\text{f}} = \bar{s}_{\text{f}}$ was determined by fitting the residual glucose concentration profile in the oxygen-limited regime to the one-parameter linear equation (25). The fits of the dissolved oxygen and glucose concentration profiles in Fig. 1 show that both $\underline{s}_{\text{f}}$ and $\bar{s}_{\text{f}}$ decrease when *D* increases from 0.07 to 0.20 h^−1^ at fixed *k*_l_*a* ≈ 50 h^−1^, and these trends are illustrated in Fig. 3a by the open and closed circles, respectively. The fits of the dissolved oxygen and glucose concentration profiles in Fig. 2 show that both $\underline{s}_{\text{f}}$ and $\bar{s}_{\text{f}}$ increase when *k*_l_*a* increases from 50 h^−1^ to 100 h^−1^ at fixed *D* = 0.1 h^−1^, a trend illustrated in Fig. 3a by the open and closed triangles, respectively.

The variations of $\underline{s}_{\text{f}}$ and $\bar{s}_{\text{f}}$ in Fig. 3a are consistent with the model. Indeed, Eqs. (17) and (26) can be rewritten as $D\underline{s}_{\text{f}} = Y_{\text{os}} k_{\text{l}} a \cdot c_{\text{o}}^{*}$ and $D\bar{s}_{\text{f}} = Y_{\text{os}}^{\prime } \cdot k_{\text{l}} a \cdot c_{\text{o}}^{*}$, and it follows that

1. When *k*_l_*a* and $c_{\text{o}}^{*}$ are fixed, $\underline{s}_{\text{f}}$ and $\bar{s}_{\text{f}}$ are inversely proportional to *D* (solid and dashed curves in Fig. 3a), a result which agrees with the experimentally determined values of $\underline{s}_{\text{f}}$ and $\bar{s}_{\text{f}}$ at *k*_l_*a* ≈ 50 h^−1^ (open and closed circles in Fig. 3a) Stated differently, given any $k_{\text{l}} a \cdot c_{\text{o}}^{*}$, the lower and upper boundaries of dual-limited growth are completely determined by the glucose influx rate—they are given by the equations $D\underline{s}_{\text{f}} = 0.47$ g L^−1^ h^−1^ and $D\bar{s}_{\text{f}} = 3.2$ g L^−1^ h^−1^. Consequently, the dashed and solid curves of Fig. 3a reduce to points on the $k_{\text{l}} a \cdot c_{\text{o}}^{*}$ − *Ds*_f_ plane (open and closed circles in Fig. 3b).
2. When *D* and $c_{\text{o}}^{*}$ are fixed, $\underline{s}_{\text{f}}$ and $\bar{s}_{\text{f}}$ are directly proportional to *k*_l_*a*, which agrees with the experimental data, since the values of $\underline{s}_{\text{f}}$ and $\bar{s}_{\text{f}}$ obtained at *D* = 0.1 h^−1^, *k*_l_*a* ≈ 50 h^−1^ (open and closed circles in Fig. 3a) are half the values of $\underline{s}_{\text{f}}$ and $\bar{s}_{\text{f}}$ obtained at *D* = 0.1 h^−1^, *k*_l_*a* ≈ 100 h^−1^ (open and closed triangles in Fig. 3a). The boundaries corresponding to the latter experiment appear on the $k_{\text{l}} a \cdot c_{\text{o}}^{*}$ − *Ds*_f_ plane as points, represented by open and closed triangles in Fig. 3b, with double the coordinates corresponding to the former experiment.

Thus, we have shown that the observed boundaries of the dual-limited regime shown in Fig. 3a agree quantitatively with those predicted by the model, and these boundaries can be represented more concisely on the $k_{\text{l}} a \cdot c_{o}^{*}$ − *Ds*_f_ plane (Fig. 3b)—the lower boundary is the line $Ds_{\text{f}} = Y_{\text{os}} k_{\text{l}} a \cdot c_{\text{o}}^{*}$ separating the carbon- and dual-limited regimes, and the upper boundary is the line $Ds_{\text{f}} = Y_{\text{os}}^{\prime } k_{\text{l}} a \cdot c_{\text{o}}^{*}$ separating the dual- and oxygen-limited regimes.

It follows from Fig. 3b that the dual-limited regime is characterized by the relation $Y_{\text{os}} < Ds_{\text{f}} /\left( {k_{\text{l}} a \cdot c_{\text{o}}^{*} } \right) < Y_{\text{os}}^{\prime }$. In earlier work, we showed that this characterization was plausible, because in the dual-limited regime, $Ds_{\text{f}} /\left( {k_{\text{l}} a \cdot c_{\text{o}}^{*} } \right)$ equals the ratio of glucose to oxygen consumption rates, which increases from the value *Y*_os_ at the left boundary coinciding with the carbon-limited regime to the value $Y_{\text{os}}^{\prime }$ at the right boundary coinciding with the oxygen-limited regime [20]. However, the characterization was experimentally validated only at *D* = 0.1 h^−1^ and *k*_l_*a* ≈ 50 h^−1^. We have now shown that the characterization is valid over the range $0.07 \;{\text{h}}^{ - 1} \le D \le 0.20 \;{\text{h}}^{ - 1}$ and $50 \;{\text{h}}^{ - 1} \le k_{l} a \le 100 \;{\text{h}}^{ - 1}$.

### The steady-state concentrations are completely determined by the ratio $Ds_{\text{f}} /\left( {k_{\text{l}} a \cdot c_{\text{o}}^{*} } \right)$

We have shown above that the boundaries of the dual-limited regime are determined the dimensionless ratio:

34 $$\rho \equiv \frac{{Ds_{\text{f}} }}{{k_{\text{l}} a \cdot c_{\text{o}}^{*} }} = \frac{{{\text{Maximum}}\;{\text{glucose consumption rate}}}}{\text{Maximum oxygen consumption rate}}.$$

Since the boundaries of the dual-limited limited regime are ultimately determined by the points at which the concentrations of dissolved oxygen and residual glucose become vanishingly small, it seems plausible that the boundaries are determined by *ρ* because these concentrations are determined by *ρ*. We show below that the model implies, and the experiments confirm, that the concentrations of not only dissolved oxygen and glucose, but also biomass and ethanol, are completely determined by *ρ*, provided the concentrations are suitably scaled.

To this end, define the dimensionless concentrations:

35 $$\sigma \equiv \frac{s}{{s_{\text{f}} }}, \;\;\omega \equiv \frac{{c_{\text{o}} }}{{c_{\text{o}}^{*} }},\;\; \chi \equiv \frac{Dx}{{k_{\text{l}} a \cdot c_{\text{o}}^{*} }}, \;\;\pi \equiv \frac{Dp}{{k_{\text{l}} a \cdot c_{\text{o}}^{*} }}.$$

Then, Eqs. (13)–(16) imply that in the carbon-limited regime, the dimensionless steady-state concentrations are

36 $$\sigma \ll 1,\;\; \omega = 1 - \frac{\rho }{{Y_{\text{os}} }}, \;\;\chi = Y_{\text{sx}} \rho ,\;\; \pi = Y_{\text{sp}} \rho ,$$

and Eqs. (22)–(25) imply that in the oxygen-limited regime, the dimensionless steady-state concentrations are

37 $$\omega \ll 1, \;\;\sigma = 1 - \frac{{Y_{\text{os}}^{\prime } }}{\rho }, \;\;\chi = Y_{\text{ox}}^{\prime } , \;\;\pi = Y_{\text{op}}^{\prime } .$$

The model, therefore, implies that for a given strain, and hence given yields, the dimensionless steady-state concentrations in the carbon- and oxygen-limited regimes are completely determined by *ρ*.

**Fig. 4:**
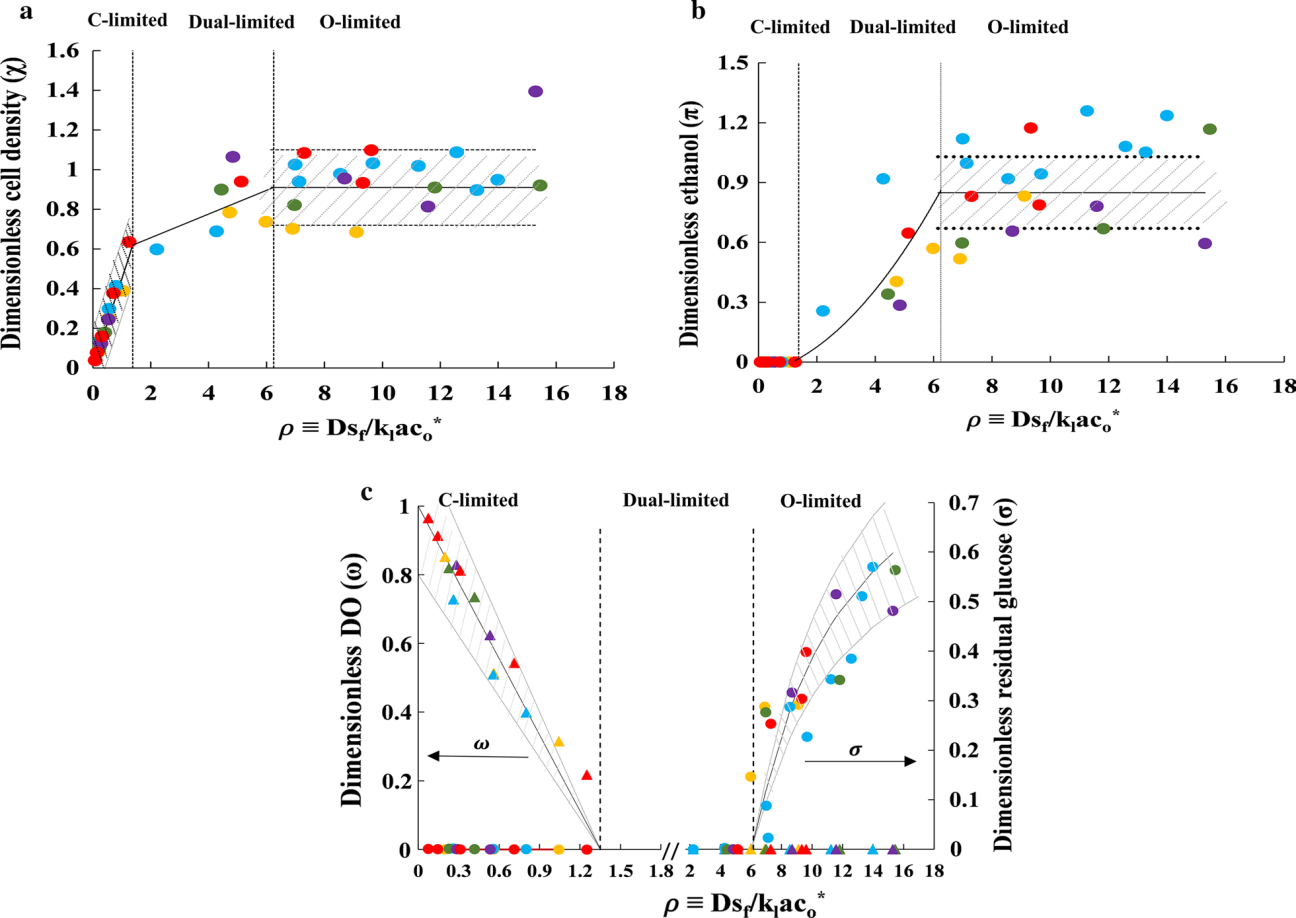
Variation of the dimensionless concentrations of **a** biomass, **b** ethanol, **c** dissolved oxygen and residual glucose with the dimensionless operating parameter *ρ*. The different symbols represent data obtained at various *k*_l_*a* and *D*, i.e., *k*_l_*a* = 50 h^−1^ and *D* =  0.07 h^−1^ (yellow), 0.10 h^−1^ (blue), 0.15 h^−1^ (green), 0.20 h^−1^ (violet); and *k*_l_*a* = 100 h^−1^ and *D* = 0.1 h^−1^ (red). The lines represent the model predictions from Eqs. (36), (37) with the experimentally determined yields shown in Table 1, and the shaded regions represent ± 20% deviation from the model predictions

Thus far, we have shown that the observed concentration profiles are consistent with the model, which in turn implies that suitably scaled concentrations are functions of *ρ*. One, therefore, expects that the measured concentrations obtained at various *D*, *s*_f_, *k*_l_*a* will also collapse into a single curve, provided that they are scaled in accordance with Eq. (35), and plotted against the dimensionless parameter *ρ* calculated from measured values of the operating parameters. Figure 4 shows that this is indeed the case, and the trends agree well with those predicted by Eqs. (36), (37) with the experimentally measured yields shown in Table 1. In the carbon-limited regime, the dimensionless biomass concentration $\chi$ increases linearly with *ρ* (Fig. 4a), the dimensionless ethanol concentration *π* is negligible (Fig. 4b), and the dimensionless dissolved oxygen level *ω* decreases linearly with *ρ* (Fig. 4c). In the oxygen-limited regime, the data are considerably more scattered for reasons discussed below, but the general trend is clear—the dimensionless ethanol and biomass concentrations, *π* and *χ*, are approximately constant (Fig. 4a, b), and the dimensionless glucose concentration *σ* increases, albeit non-linearly (Fig. 4c). Importantly, even though the operating parameters *D*, *k*_l_*a*, and *s*_f_ were varied several fold, most of the dimensionless concentrations are within 20% of the values predicted by Eqs. (36), (37) with no adjustable parameters, since all the yields were obtained from experimental data (Table 1).

The data are more scattered in the dual- and oxygen-limited regimes due to the occurrence of excessive foaming despite the use of automatic foam control, a problem that has also been reported by other researchers [21, 22]. The foaming also led to a marked decline in the closure of the carbon balance (Additional file 1: Figure S3). Our recent studies suggest that the usual strategy of adding anti-foam in response to foam formation is not effective. It is better to add anti-form periodically, thus preventing the formation of foam, rather than attempting to control it after it has formed [23].

## Discussion

### Kinetics of dual-limited regime

We have shown above that the dimensionless concentrations in the carbon- and oxygen-limited regimes are completely determined by the dimensionless parameter *ρ*. It is relevant to ask if this conclusion is also valid in the dual-limited regime.

It is evident that our models of carbon- and oxygen-limited growth are not valid in the dual-limited regime. Indeed, these models represent growth as single quasi-reactions which imply that the ratio of reaction rates of any two species such as glucose and oxygen are constant (*r*_s_/*r*_o_ = *Y*_os_, ${{r_{\text{s}}^{\prime } } \mathord{\left/ {\vphantom {{r_{\text{s}}^{\prime } } {r_{\text{o}}^{\prime } }}} \right. \kern-0pt} {r_{\text{o}}^{\prime } }} = Y_{\text{os}}^{\prime }$), but the dual-limited regime is characterized by increasing ratio of glucose to oxygen consumption rates $Ds_{\text{f}} /\left( {k_{\text{l}} a \cdot c_{\text{o}}^{*} } \right)$. Although our models are not valid in the dual-limited regime, the dimensionless variables are still well-defined and physically meaningful. In particular, $\chi \equiv Dx/\left( {k_{\text{l}} a \cdot c_{\text{o}}^{*} } \right)$ and $\pi \equiv Dp/\left( {k_{\text{l}} a \cdot c_{\text{o}}^{*} } \right)$ are approximately equal to the yields of biomass and ethanol on oxygen. Figurse 4a, b, which suggest that *χ* and *π* are functions of *ρ* in the dual-limited regime, therefore, implies that the yields of biomass and ethanol are functions of *ρ*. This result is consistent with the literature [24]. Indeed, von Stockar and Birou formulated a mathematical model which assumes that the kinetics of the dual-limited regime are the outcome of not one, but two, independent quasi-reactions, namely, respiration and fermentation [24]. The model implies that in the dual-limited regime, the biomass and ethanol yields are functions of the aerobicity $\varOmega \equiv k_{\text{l}} a \cdot c_{\text{o}}^{*} /\left( {Ds_{\text{f}} Y_{\text{so}} } \right) = 1/\left( {\rho Y_{\text{so}} } \right)$, and hence also functions of *ρ*, which is consistent with the data in Fig. 4a, b. Their experiments with *Kluyveromyces fragilis* also confirmed that the variation of the observed biomass and ethanol yields with *Ω* agreed with their model predictions. However, we could not determine if our data are quantitatively consistent with their model, since it requires anaerobic growth yields, but *S. stipitis* grows very poorly under anaerobic conditions. We are currently acquiring more data in the dual-limited regime, and exploring methods for extracting the parameters of fermentative growth without subjecting the cells to anaerobic growth.

### Implications of the existence of the dimensionless plot

The existence of the dimensionless plot provides a powerful tool for comparing the parametric sensitivities of different strains. Indeed, since the effects of *D*, *s*_f_, *k*_l_*a*, and $c_{\text{o}}^{*}$ are embedded in the single parameter *ρ*, superimposing the dimensionless plots for two strains immediately reveals not only the range of operating parameters that support ethanol production without loss of the carbon source, but also their performance as ethanol producers. The dimensionless plot would be particularly useful for comparing *S. stipitis* with the benchmark strain *Saccharomyces cerevisiae*, an effort that is already under way in our lab.

Although the dimensionless plot is a powerful tool for comparing parametric sensitivity of strains, it is of limited use if it requires extensive data at multiple *D*, *s*_f_, *k*_l_*a*, and $c_{\text{o}}^{*}$. It is, therefore, convenient that the very existence of the dimensionless plot implies that it can be generated by only a few experiments. For instance, we can measure the steady-state concentrations at any one set of fixed *D* and *k*_l_*a* (Figs. 1 and 2), but if we plot these data in the dimensionless form (Fig. 4), the graph obtained captures the data over an entire range of the operating parameters. In fact, it is not necessary to hold *D* and *k*_l_*a* at fixed values—any combination of *D* and *k*_l_*a* can be used to generate the dimensionless plot, since the variations of these parameters are automatically normalized by the dimensionless concentrations and parameter *ρ*. This is useful, since it is practically quite difficult to maintain constant values of *k*_l_*a*. Indeed, the values of *k*_l_*a* attained in our experiments differed from the desired values of 50 h^−1^ and 100 h^−1^ by up to ± 15%, but the dimensionless plot automatically corrects for these variations. Thus, only one set of data, even if it is obtained at varying *D* and *k*_l_*a*, provides information about the steady states obtained over a range of operating conditions.

### Comparison with data in the literature

**Fig. 5:**
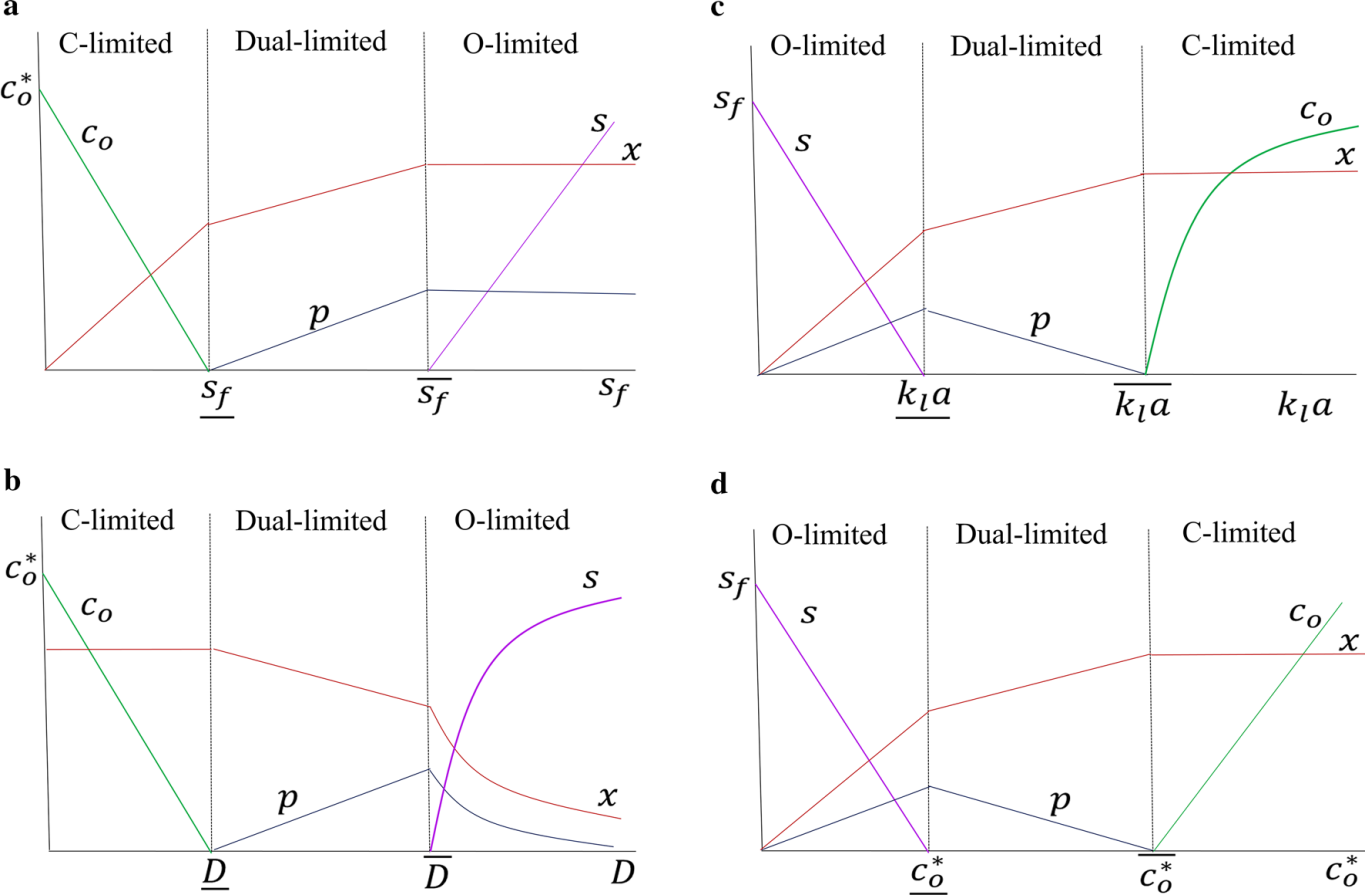
Schematic diagrams illustrating the predicted steady-state concentration profiles of biomass *x*, ethanol *p*, dissolved oxygen *c*_o_ and residual carbon source *s* in continuous cultures operated, such that **a** *s*_f_ is varied at fixed *D*, *k*_l_*a*, $c_{\text{o}}^{*}$, **b** *D* is varied at fixed *s*_f_, *k*_l_*a*, $c_{\text{o}}^{*}$, **c** *k*_l_*a* is varied at fixed *D*, *s*_f_, $c_{\text{o}}^{*}$, **d** $c_{\text{o}}^{*}$ is varied at fixed *D*, *s*_f_, *k*_l_*a*. The concentration profiles in the carbon- and oxygen-limited regimes are based on Eqs. (13)–(16) and (22)–(25), respectively. The concentration profiles in the dual-limited regime are obtained by linearly interpolating the end points of the concentration profiles in the carbon- and oxygen-limited regimes

In our experiments, the feed concentration *s*_f_ was varied at fixed *D*, *k*_l_*a*, and $c_{\text{o}}^{*}$ which led to the concentration profiles shown schematically in Fig. 5a. In contrast, all the experiments reported in the literature were performed such that *D*, *k*_l_*a*, or $c_{\text{o}}^{*}$ was varied, and the remaining three parameters were held fixed. Since Eqs. (13)–(16) and (22)–(25) capture the variation of the steady states even when *D*, *k*_l_*a*, or $c_{\text{o}}^{*}$ are varied (Fig. 5b–d), we can compare the data in the literature with the model predictions.

### $D$ varied at fixed $s_{\text{f}}$, $k_{\text{l}} a$, and $c_{\text{o}}^{*}$

In this case, the model predicts the profile shown in Fig. 5b. In the carbon-limited regime, the cell density remains constant, and the dissolved oxygen concentration declines linearly to sub-critical levels. In the oxygen-limited regime, the concentration of the carbon source increases non-linearly, and the concentrations of biomass and ethanol are inversely proportional to *D*.

We are aware of only one study in which this class of experiments was performed, and these data are consistent with the picture shown in Fig. 5b. Slininger et al. reported the variation of the biomass concentration with the dilution rate in oxygen-limited continuous cultures of *S. stipitis* operated at various *k*_l_*a* ranging from 1.0 to 4.6 h^−1^ [15]. The biomass concentrations declined with the dilution rate in a manner consistent with the picture in Fig. 5b. In fact, the equation describing the variation of the biomass concentration derived by Slininger et al. reduces to Eq. (23) if one neglects cell death and loss of dissolved oxygen via the effluent liquid stream.

### $k_{\text{l}} a$ varied at fixed $D$, $s_{\text{f}}$, and $c_{\text{o}}^{*}$

In this case, the model predicts that the oxygen- and carbon-limited regimes occur at small and large *k*_l_*a*, respectively (Fig. 5c). In the oxygen-limited regime, the biomass and product concentrations increase linearly, and the residual carbon source concentration decreases linearly. In the carbon-limited regime, the biomass and ethanol levels are constant, and the dissolved oxygen level increases non-linearly.

We are aware of only one study in which this class of experiments was performed. Grootjen et al. varied *k*_l_*a* at fixed *D*, *s*_f,_ and $c_{\text{o}}^{*}$ in steady-state continuous cultures of *S. stipitis*, but they did not report the variation of the concentrations with *k*_l_*a* [8]. Instead, they reported the variation of the glucose consumption rate, the biomass formation rate, and ethanol formation rate with the oxygen consumption rate. However, we can still compare their data to Fig. 5c, because *D* was constant in their experiments, which implies that the glucose consumption rate *D*(*s*_f_ − *s*), biomass formation rate *Dx*, and ethanol formation rate *Dp* are proportional to *s*_f_ − *s*, *x*, and *p*, respectively. Moreover, since ethanol was produced even at the highest oxygen consumption rates reported in their experiments, their data were obtained under oxygen- or dual-limited conditions under which the oxygen consumption rate is approximately $k_{\text{l}} a\left( {c_{\text{o}}^{*} - c_{\text{o}} } \right) \approx k_{\text{l}} a \cdot c_{\text{o}}^{*}$ which is proportional to *k*_l_*a*, since $c_{\text{o}}^{*}$ was constant. Given these facts, their data are consistent with Fig. 5c: At low oxygen consumption rates, *D*(*s*_f_ − *s*), *Dx* and *Dp* increased linearly with the oxygen consumption rate $k_{\text{l}} a \cdot c_{\text{o}}^{*}$ until glucose was exhausted, and beyond this point, *Dx* increased and *Dp* decreased in a manner consistent with the trends for the dual-limited regime.

### $c_{\text{o}}^{*}$ varied at fixed $D$, $s_{\text{f}}$, and $k_{\text{l}} a$

In this case, the model predicts concentration profiles that are formally identical to those obtained when *k*_l_*a* is varied, the only difference being that $c_{\text{o}}$ increases linearly in the carbon-limited regime (Fig. 5d).

To our knowledge, this class of experiments has only been performed with microbes such as *K. aerogenes* and *K. fragilis*, that, unlike Crabtree-negative yeasts, grow and ferment even under anaerobic conditions [24–26]. Interestingly, there is no evidence of oxygen-limited growth in these studies, since growth is dual-limited when $c_{\text{o}}^{*}$  = 0. Indeed, glucose is completely consumed when $c_{\text{o}}^{*}$ = 0, and when $c_{\text{o}}^{*}$ increases beyond zero, the biomass concentration increases, and the fermentation product concentration(s) decreases in a manner consistent with the trends expected from the dual-limited, rather than the oxygen-limited, regime (Fig. 5d). It follows that if such strains are studied by varying *s*_f_ at fixed *D*, *k*_l_*a*, and $c_{\text{o}}^{*}$ (Fig. 5a), the oxygen-limited regime will not be attained even at arbitrarily large *s*_f_, an intriguing result that remains to be verified.

We note finally that Weusthuis et al. studied the steady states attained by *Candida utilis* and *S. cerevisiae* by varying both *s*_f_ and $c_{\text{o}}^{*}$ simultaneously [24–26], an experiment that is easier to visualize by appealing to Fig. 3b. Despite the operational difference, their data agreed with those described above. The Crabtree-negative yeast *C. utilis* yielded carbon-, dual-, and oxygen-limited regimes, but the oxygen-limited regime was not observed with *S. cerevisiae*, which, like *K. fragilis* and *K. aerogenes*, grows well anaerobically.

Taken together, the above analysis shows that our model predictions are consistent with experiments in which *D*, *k*_l_*a*, or $c_{\text{o}}^{*}$ (rather than *s*_f_) was varied. Moreover, we have confined attention to studies concerned primarily with ethanol production, but it might be useful to examine the existence of the scaling relation in the production of other compounds such as 2,3-butanediol, since the kinetics of these processes agree with our model [25].

## Conclusions

Recently, we showed that when a continuous culture of *S. stipitis* operated at *D* = 0.1 h^−1^ and *k*_l_*a* ≈ 50 h^−1^ was fed with progressively higher concentrations of glucose, the transition from carbon- to oxygen-limited growth occurred via a well-defined intermediate region of dual-limited growth, characterized by the relation $Y_{\text{os}} < Ds_{\text{f}} /\left( {k_{\text{l}} a \cdot c_{\text{o}}^{*} } \right) < Y_{\text{os}}^{\prime }$, in which ethanol was produced without loss of glucose [20]. Here, we wished to check if the above characterization is valid over a range of operating conditions and to understand why the dual-limited regime is determined by the dimensionless ratio $\rho \equiv Ds_{\text{f}} /\left( {k_{\text{l}} a \cdot c_{\text{o}}^{*} } \right)$. To this end, we performed continuous culture experiments at three additional *D* and one additional *k*_l_*a*. We found that

1. The above characterization of the dual-limited regime is valid over the range $0.07\;{\text{h}}^{ - 1} \le D \le 0.20\;{\text{h}}^{ - 1}$ and $50\;{\text{h}}^{ - 1} \le k_{\text{l}} a \le 100\;{\text{h}}^{ - 1}$.
2. The boundaries of the dual-limited regime are determined by the dimensionless parameter *ρ* because more generally, all the steady-state concentrations, when suitably scaled, are determined by *ρ*. The boundaries of the dual-limited regime are merely special points of the dimensionless oxygen and residual glucose concentrations, namely, the points at which these concentrations intersect the *ρ*-axis.
3. The existence of the dimensionless relation provides a powerful tool for systematically comparing different ethanologenic strains without significant acquisition of data.

## Methods

### Strain and media

Wild-type *S. stipitis* CBS 6054 was used in all experiments. The yeast was maintained on solid YPD (yeast extract peptone dextrose) medium at 4 °C for regular use. All the experiments were done with glucose as carbon source in defined medium described previously [20]. The maximum specific growth rate *μ*_max_ of *S. stipitis* in the defined minimal medium is 0.45 ± 0.03 h^−1^ (Table S2, Supplementary, [20]).

### Chemostat setup

The reactor design and operating conditions were the same as mentioned previously [20]. The impeller speed was varied between 500 and 700 rpm to maintain *k*_l_*a* in a definite range (45–55 h^−1^, 95–110 h^−1^). Online profiles of the dissolved oxygen, temperature, pH, impeller speed, and off-gas (O_2_ and CO_2_) were obtained from the Applikon control unit using BioXpert software. For data analysis, three steady-state samples were taken at an interval of 6–8 h at each feed glucose concentration.

### Chemostat cultivation conditions

A colony of pure *S. stipitis* culture was grown overnight in YPD (1% yeast extract 2% peptone 2% dextrose) liquid medium at 30 °C in a shake flask. 1 mL of this primary culture was washed and inoculated in another shake flask containing 500 mL of defined minimal medium the same as used in bioreactor. The secondary culture was grown for 6–8 doublings. An aliquot (5–10 mL) of secondary culture was inoculated aseptically into reactor vessel with a working volume of 1 L to obtain an initial optical density (OD_600_) of 0.1. The reactor vessel was maintained at 30 °C before inoculation. 1 mL of sterile anti-foaming agent was added to prevent foaming during batch culture. The cells were grown in batch until late log phase. Chemostat mode was initiated by connecting feed and outlet pump to the reactor vessel.

### Estimation of biomass concentration

A known volume of culture was washed twice with water and filtered through a cellulose nitrate membrane filter (0.2 μm) with the help of a vacuum pump. The filtered cells were dried at 80 °C until the mass became constant. The detection limit of Sartorius Analytical balance used for weighing filtered cells was 0.1 mg with a repeatability of ± 0.3 mg.

### Estimation of sugar and fermentation products

Fermentation broth was filtered and analyzed for sugar and fermentation products. Glucose was estimated by high-performance liquid chromatography (HPLC). With standards, the lowest concentration of glucose detected by HPLC system was 5 mg L^−1^, but with fermentation broth, the background noise was too large for precise measurement of the glucose concentrations, specifically, when the samples had less than 80 mg L^−1^ of glucose. A given sample was varied by ± 0.05 g L^−1^ on multiple injection. Ethanol and acetic acid were analyzed by gas chromatography (GC-FID). The limit of detection for ethanol and acetic acid was 0.05 g L^−1^ and 0.005 g L^−1^, respectively, with a repeatability of ± 0.5 mg L^−1^ for ethanol and ± 0.1 mg L^−1^.The analysis was done using the same equipment and conditions as those described previously [20].

### Determination of mass-transfer coefficient $k_{\text{l}} a$ and specific uptake rate $r_{\text{o}}$ of oxygen

Two distinct methods were used for the analysis under carbon-limited regime and oxygen-limited regime. Dynamic out-gassing and steady-state difference methods were used for carbon- and oxygen-limited regimes, respectively, as reported previously [20]. The details of the two methods are given below:

#### Dynamic out-gassing method

For determining *k*_l_*a* of the culture growing at steady state, the air supply of the culture growing at steady state was stopped briefly. Specifically, the dissolved oxygen level was allowed to fall to no less than 30–35% of saturation, so that the cells do not experience oxygen limitation. As soon as the DO level reached 30–35%, the air supply was resumed. The DO level then increases gradually and stabilizes at steady-state level. The initial linear decrease of the DO to 30–35% yields the oxygen uptake rate, and the later temporal increase of DO yields *k*_l_*a* and $c_{\text{o}}^{*}$ (Figure S1, Supplementary [20]).

#### Steady-state method

The steady-state method is based on the mass balance for oxygen in the air stream

$$k_{\text{l}} a \cdot c_{\text{o}}^{*} \left( {1 - \frac{{c_{\text{o}} }}{{c_{\text{o}}^{*} }}} \right) = F_{{{\text{g}},{\text{in}}}} y_{{{\text{o}},{\text{in}}}} - F_{{{\text{g}},{\text{out}}}} y_{{{\text{o}},{\text{out}}}} ,$$

where *F*_g,in_, *F*_g,out_ are the molar flow rates of the inlet and outlet air streams, and *y*_o,in_, *y*_o,out_ are the mole fractions of oxygen in the inlet and outlet air streams. In the dual- and oxygen-limited regimes, *c*_o_ ≪  $c_{\text{o}}^{*}$ and measurement of *F*_g,in_, *F*_g,out_, *y*_o,in_, *y*_o,out_ yields $k_{\text{l}} a \cdot c_{\text{o}}^{*}$. To calculate *k*_l_*a*, we assumed that the value of $c_{\text{o}}^{*}$ in dual- and oxygen-limited regime was the same as that determined in the carbon-limited regime.

## Additional file

**Additional file 1.** Additional figures.

## Abbreviations

C-limited: carbon-limited
*c*_o_: steady-state concentration of dissolved oxygen (g L^−1^)
$c_{\text{o}}^{*}$: solubility of oxygen (g L^−1^)
*D*: dilution rate (h^−1^)
*F*_g,in_, *F*_g,out_: molar flow rates of the inlet and outlet air streams
*k*_l_*a*: mass-transfer coefficient of oxygen (h^−1^)
*K*_o_: critical dissolved oxygen level (mg L^−1^)
*K*_s_: saturation constant for glucose (mg L^−1^)
O-limited: oxygen-limited
*p*: steady-state concentration of ethanol (g L^−1^)
*r*_o_, $r_{\text{o}}^{\prime }$: steady-state-specific oxygen consumption rate in C- and O-limited regime (g gdw^−1^ h^−1^)
*r*_p_, $r_{\text{p}}^{\prime }$: steady-state-specific ethanol productivity in C- and O-limited regime (g gdw^−1^ h^−1^)
*r*_s_, $r_{\text{s}}^{\prime }$: steady-state-specific glucose consumption rate in C- and O-limited regime (g gdw^−1^ h^−1^)
*s*: steady-state residual glucose concentration (g L^−1^)
*s*_f_: feed glucose concentration (g L^−1^)
$\underline{s}_{\text{f}}$, $\bar{s}_{\text{f}}$: lower and upper boundaries of dual-limited regime (g L^−1^)
*t*: time (h)
*x*: steady-state biomass concentration (gdw L^−1^)
*y*_o,in_, *y*_o,out_: mole fractions of oxygen in the inlet and outlet air streams
*Y*_op_, $Y_{\text{op}}^{\prime }$: yield of ethanol on oxygen in C- and O-limited regime (g g^−1^)
*Y*_ox_, $Y_{\text{ox}}^{\prime }$: yield of biomass on oxygen in C- and O-limited regime (gdw g^−1^)
*Y*_sc_, $Y_{\text{sc}}^{\prime }$: CO_2_ produced per g of glucose in C- and O-limited regime (g g^−1^)
*Y*_so_, $Y_{\text{so}}^{\prime }$: oxygen consumed per g of glucose in C- and O-limited regime (g g^−1^)
*Y*_sp_, $Y_{\text{sp}}^{\prime }$: ethanol produced per g of glucose in C- and O-limited regime (g g^−1^)
*Y*_sx_, $Y_{\text{sx}}^{\prime }$: biomass produced per g of glucose in C- and O-limited regime (g gdw^−1^)
*μ*, *μ*^′^: specific growth rates in C- and O-limited regime (h^−1^)
*μ*_m_: maximum specific growth rate (h^−1^)
*π*: steady-state dimensionless ethanol concentration
*ρ*: steady-state dimensionless ratio of operating parameters
*σ*: steady-state dimensionless concentration of residual glucose
*χ*: steady-state dimensionless concentration of biomass
*ω*: steady-state dimensionless concentration of dissolved oxygen
