# Supplementary material for: Existence of a scaling relation in continuous cultures of Scheffersomyces stipitis: the steady states are completely determined by the ratio of carbon and oxygen uptake rates
Source: Biotechnol Biofuels. 2019 Jan 28;12:19. doi: 10.1186/s13068-019-1357-3 (PMC6348663; doi:10.1186/s13068-019-1357-3)
Supplement: Supplementary file 1 — Additional file 1. Additional figures. [file 13068_2019_1357_MOESM1_ESM.pdf]

Additional file 1

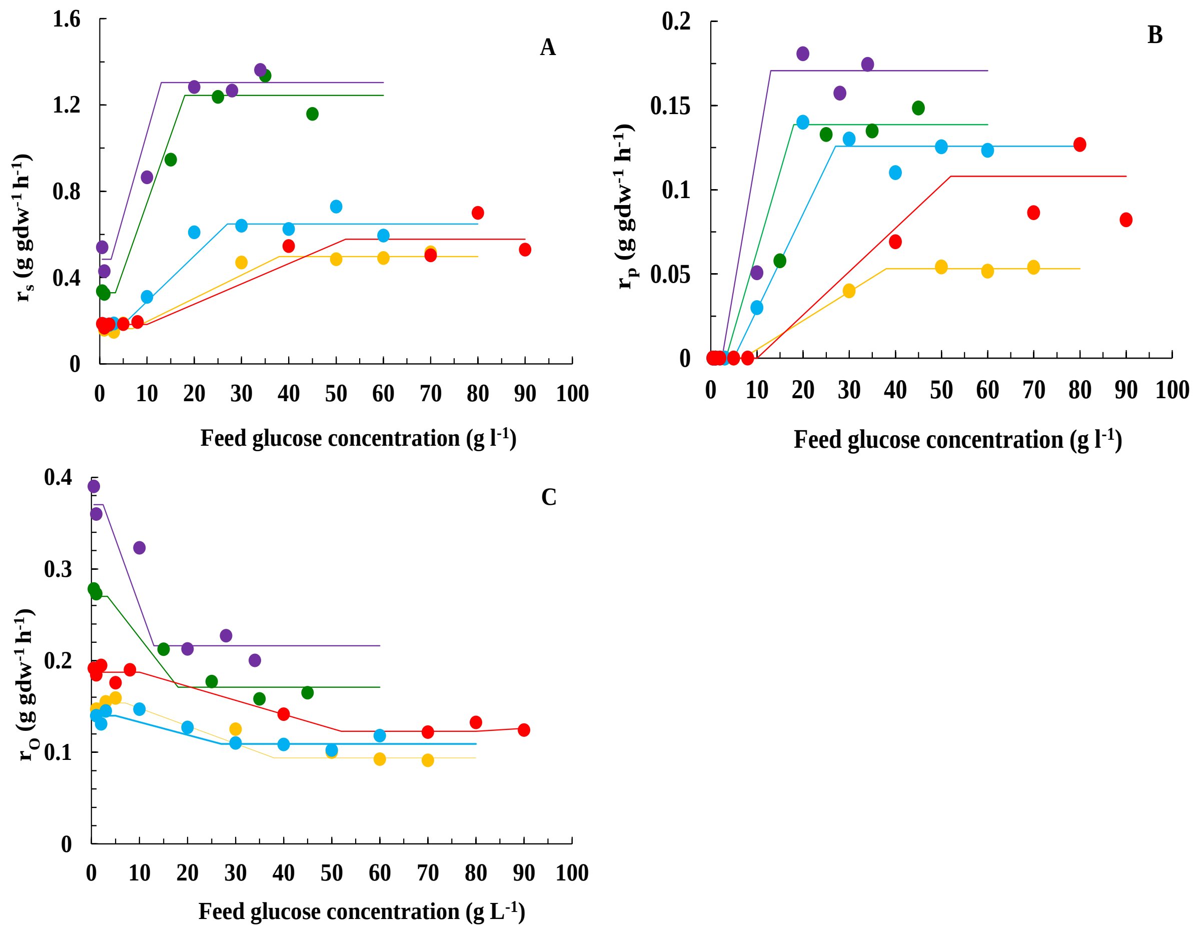

Figure S1: Variation of the steady state specific rates of glucose consumption rate r_s, ethanol production r_p, and oxygen consumption r_o as a function of the concentration of glucose fed to a chemostat. The specific rates in the carbon- and oxygen-limited regimes are independent of the feed glucose concentration, but increase with the dilution rate. The different symbols represent the various operating conditions: $k_l a \approx 50$ h^{-1} and $D = 0.07$ h^{-1} (●), 0.10 h^{-1} (●), 0.15 h^{-1} (●), 0.20 h^{-1} (●); and $k_l a \approx 100$ h^{-1} and $D = 0.1$ h^{-1} (●).

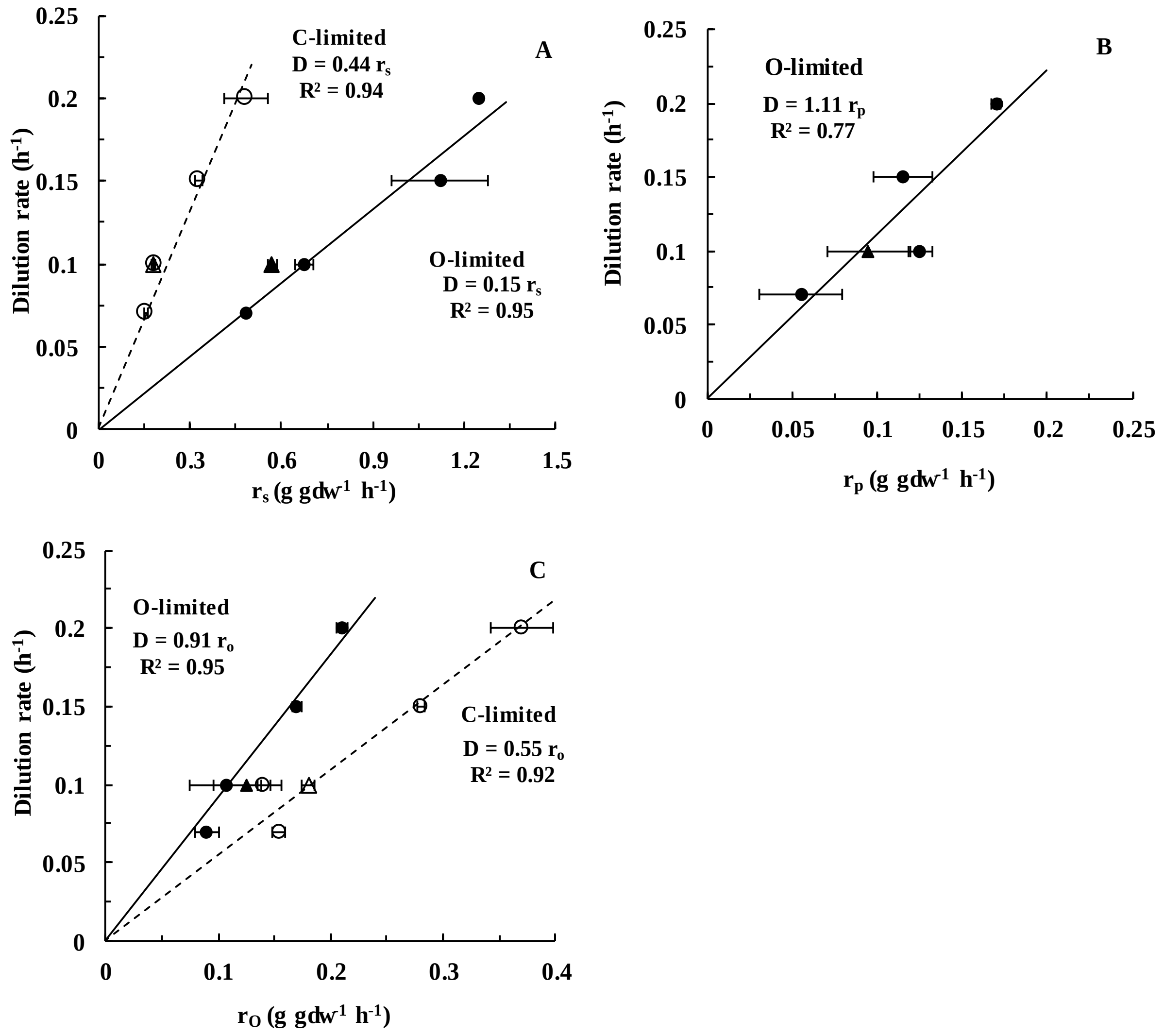

Figure S2: Determination of the biomass yields in the carbon- and oxygen-limited regimes. (A) Yields of biomass on glucose are $Y_{sx} = 0.44$ gdw g^{-1} and $Y'_{sx} = 0.15$ gdw g^{-1} (B) Yields of biomass on ethanol are $Y'_{px} = 1.11$ gdw g^{-1} (C) Yields of biomass on oxygen are $Y_{ox} = 0.55$ gdw g^{-1} and $Y'_{ox} = 0.91$ gdw g^{-1}. The data for $k_l a \approx 50$ h^{-1} and 100 h^{-1} are represented by circles and triangles, respectively. The data for carbon- and oxygen-limited regimes are represented by open and closed symbols, respectively. The fits to these data are represented by dashed and solid lines, respectively.

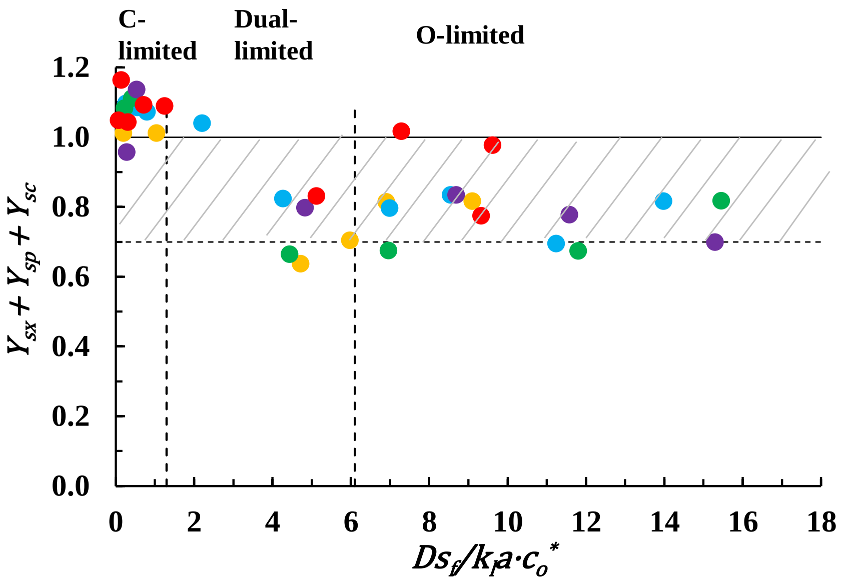

Figure S3: Closure of the carbon balance in carbon-, dual-, and oxygen-limited regimes assuming that the cell composition is $CH_{1.61}O_{0.5}N_{0.16}$. In the carbon-limited regime $0 < Ds_f/(k_l a \cdot c_o^*) < 1.3$, the carbon balances close well since $Y_{sx} + Y_{sp} + Y_{sc} \approx 1.1$. However, in the dual- and oxygen-limited regimes $Ds_f/(k_l a \cdot c_o^*) > 6.1$, the balances do not close well since the sum of the yields is 0.7-0.8. The poorer carbon recovery in these regimes is due to excessive foaming which does not occur in the carbon-limited regime. The shaded region represents 30 % deviation from complete carbon recovery. The different symbols represent the various operating conditions, $k_l a = 50$ h^{-1} and $D = 0.07$ h^{-1} (●), 0.10 h^{-1} (●), 0.15 h^{-1} (●), 0.20 h^{-1} (●); $k_l a = 100$ h^{-1} and $D = 0.1$ h^{-1} (●).
